# Clinical performance and occlusal wear of polymer-infiltrated ceramic network and zirconia-reinforced ceramic implant-supported single crowns fabricated via a digital workflow on two titanium implant systems: a 12-month prospective randomized trial

**DOI:** 10.1186/s40729-026-00671-9

**Published:** 2026-02-20

**Authors:** Malte Bagratuni, Maria Bruhnke, Lauren Bohner, Insa Herklotz, Florian Beuer, Mats Wernfried Heinrich Böse, Stefano Pieralli

**Affiliations:** 1 Department of Prosthodontics, Geriatric Dentistry and Craniomandibular Disorders, Charité – Universitätsmedizin Berlin, Berlin, Germany; 2 Department of Oral and Maxillofacial Surgery, Universitätsklinikum Münster, Münster, Germany; 3 Private Dental Office, Berlin, Germany

**Keywords:** Implant, Digital workflow, Ceramics, Survival rate, Polymer-infiltrated ceramic network, Zirconia-reinforced lithium silicate ceramic

## Abstract

**Purpose:**

Polymer-infiltrated ceramic network (PICN) and zirconia-reinforced lithium silicate (ZLS) ceramics are alternatives for implant-supported single crowns (ISCs). This prospective randomized trial evaluated 12-month survival of PICN- and ZLS-ISCs supported by Straumann Bone Level Tapered (BLT) and CAMLOG Promote plus (PP) titanium implants. Furthermore, technical complications and occlusal wear were analyzed.

**Methods:**

Sixty-one patients received a single titanium implant from two different manufacturers, and one was treated with two implants. All implants were placed using fully guided static computer-assisted surgery. After randomization, screw-retained CAD-CAM ISCs were fabricated from PICN (n = 31) or ZLS (n = 31) and cemented onto stock titanium base abutments. After 1 year, implant and prosthetic survival, material- and system-dependent outcomes, technical complications, and occlusal wear—based on superimposed baseline and follow-up intraoral scans—were recorded. Independent t-tests were performed (p = 0.05).

**Results:**

Forty-two ISCs were evaluated after one year (mean follow-up: 409.5 ± 102.6 days). PICN showed a 84% survival rate, while ZLS achieved 100% survival. Debonding occurred in five restorations (PICN = 2; ZLS = 3). No complications were observed in 86% of PICN and 85% of ZLS crowns. Occlusal wear was significantly greater for PICN (0.45 ± 0.38 mm) compared with ZLS (0.16 ± 0.05 mm; p = 0.03). Both crown fractures occurred on BLT implants (82% survival), whereas PP implants showed 100% survival.

**Conclusions:**

ZLS demonstrated the highest survival rate and no irreparable failures, along with significantly lower occlusal wear. PICN showed more catastrophic fractures and greater wear. Technical complications were frequent for both materials, independent of implant system or abutment height.

**Trial registration:**

Deutsches Register Klinischer Studien (DRKS-ID: DRKS00026973).

## Background

Implant-supported single crowns (ISCs) are increasingly being applied as an alternative to fixed dental prostheses (FDP) or removable partial dentures [1–3]. For ISCs, ceramic materials are used either to veneer a metal or a high-performance ceramic framework or in a monolithic form [4–9]. Metal-ceramic ISCs are widely documented in the literature with 95.8% 5-year survival rates [10]. However, a markable incidence of technical complications, such as ceramic chipping, with an incidence of up to 3.5% over 5 years, can be observed [11]. Implant-supported all-ceramic ISCs, showed an estimated survival rate of ≥ 96.1% after three years, and no significant differences in terms of survival rate were assessed between reinforced glass‐ceramic and zirconia [1]. Chipping was still reported as the primary technical complication in veneered all-ceramic ISCs, whereas monolithic ISCs exhibited significantly lower chipping rates [12].

Besides lower complication rates, monolithic restorations also offer improved time efficiency and cost-effectiveness via computer-aided design/computer-aided manufacturing (CAD/CAM) [13, 14]. To address these demands, alternative materials specifically aimed at facilitating the digital workflow, including polymer-infiltrated ceramic networks (PICN), were introduced [15]. These materials exhibit a lower elastic modulus (30–37 GPa) [16], compared to other materials like reinforced glass–ceramics (95 GPa) [17]. The increased elasticity is supposed to compensate for the missing mobility of implants and offset mechanical stress during mastication. The resulting damping effect might decrease technical complications, like ceramic chipping or screw loosening [18]. While in-vitro studies have confirmed the enhanced mechanical properties of PICN, clinical data remains controversial [19, 20].

Alternatively, Zirconia-Reinforced Lithium Silicate Ceramic (ZLS) is a rather novel iteration of this class of materials, attempting to combine the advantageous aesthetic features of lithium silicate with the mechanical properties of zirconia restorations [19]. Contrary to a buffering effect of PICN, the addition of zirconia in ZLS is supposed to increase the fracture toughness and elastic modulus [21]. The improved mechanical resistance might improve performance under occlusal loads, especially in implant-supported restorations. The use for ISCs has been documented in various in-vitro investigations, however clinical data is still missing [22–25].

As clinical evidence on PICN and ZLS is scarce, the aim of this clinical study was, to assess the survival and technical complication rate of the two different restorative materials, for ISC after 12 months in function. Moreover, the influence of the two implant systems applied was evaluated. Finally, the occlusal wear of ISCs was quantified using initial and follow-up intraoral scan (IOS) data.

The null hypotheses were that the restorative material and implant system would not affect neither the survival rate, nor the technical complication rates, nor the occlusal wear of ISCs.

## Methods

### Study design and sample size calculation

This investigation was designed as a prospective randomized clinical trial and was approved by the local ethics committee (application number: EA4/111/19; date of approval: 06.08.2019). The study was conducted according to the latest version (2013) of the Declaration of Helsinki as a standard for medical ethics. Furthermore, it was registered in the German Clinical Trials Register (DRKS-ID: DRKS00026973) and implemented according to the CONSORT (Consolidated Standards of Reporting Trials) guidelines [26].

Sample size calculation was conducted using the Software R-Studio (R Foundation for Statistical Computing, Austria). Based on a previous study [27], the survival rate was considered to vary between 80 to 99% between materials. Calculations were performed by a two-tailed Log-Rank test with α-level at 0.05 and power = 80%.

### Participants

All patients requiring single implant placement at anterior or posterior sites, were screened at the Department of Prosthodontics, Geriatric Dentistry and Craniomandibular Disorders of Charité – University Medicine Berlin between May 2019 and July 2022.

Inclusion criteria were: ≥ 18 years, American Association of Anesthesiologists (ASA) classification I or II, requiring single implant placement and having completed the required surgical, conservative, and periodontal pretreatment prior to implant surgery. Moreover, tooth extraction had to be carried out at least twelve weeks before implant placement.

The exclusion criteria were ASA risk classification > II, chronic inflammatory processes, metabolic disorders or medications associated with bone lesions or bone healing (i. e. uncontrolled or poorly controlled diabetes), chemotherapy or radiation, and insufficient compliance. Major bone-augmentation procedures, such as external sinus floor elevation, were considered reasons for exclusion, while minor guided bone regeneration (GBR) occurring during implant installation was possible. Patients were not screened for bruxism and were consequently included despite possible parafunctional activities. Written informed consent was obtained from all participants one week prior to participation in the study.

### Implant selection and surgical protocol

After randomization, using a publicly accessible website (https://www.randomizer.org), participants were allocated to either receive a Straumann Bone Level Tapered (BLT, n = 30) (Roxolid, SLActive, Ø 3.3–4.8 mm, length 8–12 mm, Institut Straumann AG, Switzerland) or CAMLOG SCREW-LINE Promote plus (PP, n = 32) (Ø 3.3–4.3 mm, length 9–13 mm, CAMLOG Biotechnologies AG, Switzerland) implant. BLT implants were virtually planned using coDiagnostiX (Dental Wings GmbH, Germany), while SMOP (Swissmeda AG, Switzerland) was used for PP implants. All implants were installed using static computer-assisted implant surgery (sCAIS) to ensure an optimal prosthetically-driven implant position. The sCAIS procedure included a fully-guided drilling protocol and insertion of the implant through the 3D-printed (Form 3B printers, Formlabs GmbH, Germany) surgical guides, according to the respective manufacturer's protocol.

After a 12-week period of submerged healing, second-stage surgery was performed for implant exposure. In the next appointment, titanium scan bodies specific to the implant system were screwed on to the implants. The clinical implant position was then captured with an intraoral scanner (Trios3, 3Shape A/S, Denmark; software versions: 18.1.2. and 20.1.2.) and transferred to the dental laboratory.

### Manufacturing and delivery of the CAD/CAM ISCs

Restorative material assignment was randomized (https://www.randomizer.org), resulting in two groups: PICN (n = 31) and ZLS (n = 31). After modelling in a design software (3Shape, 3Shape A/S, Denmark), ISCs were fabricated from either a PICN with a ceramic–polymer structure (VITA ENAMIC, Vita Zahnfabrik H. Rauter GmbH & Co. KG, Germany) or a ZLS (VITA SUPRINITY, Vita Zahnfabrik H. Rauter GmbH & Co. KG, Germany). All ISCs were milled from either PICN or ZLS blocks with the same milling unit (N4, vhf camfacture AG, Germany). After milling, PICN restorations were finished by polishing. ZLS on the other hand required firing for crystallization. Following crystallization, ZLS ISCs were glazed and, successively, again fired.

Corresponding prefabricated titanium bases were used for both implant systems: RC/NC Variobase (height 3,5–5,5 mm, Ø 3,8–4,5 mm, Institute Straumann AG, Switzerland) and CAMLOG titanium bases (height 4,7 mm, Ø 3.3–4.3 mm CAMLOG Biotechnologies AG, Switzerland) were used to adhesively cement the ISCs. The bases were conditioned with airborne particle abrasion with Al_2_O_3_ (50 μm, 2 bar), followed by application of a coupling agent (Monobond Plus, Ivoclar Vivadent GmbH, Germany) for 60 s. The internal surfaces of the ISCs were etched with 5% hydrofluoric—acid for 60 s for PICN and 20 s for ZLS—and the coupling agent (Monobond Plus, Ivoclar Vivadent GmbH, Germany) was applied. A resin-based cement (Multilink Hybrid Abutment, Ivoclar Vivadent GmbH, Germany) was then used to adhesively lute the restoration onto the titanium abutments in the dental lab. Excess of the adhesive cement was removed under 15-fold magnification with a microscope (Mantis, Vision Engineering, United Kingdom). Fabrication and bonding of the ISCs was carried out according to the respective manufacturer’s protocols.

**Fig. 1:**
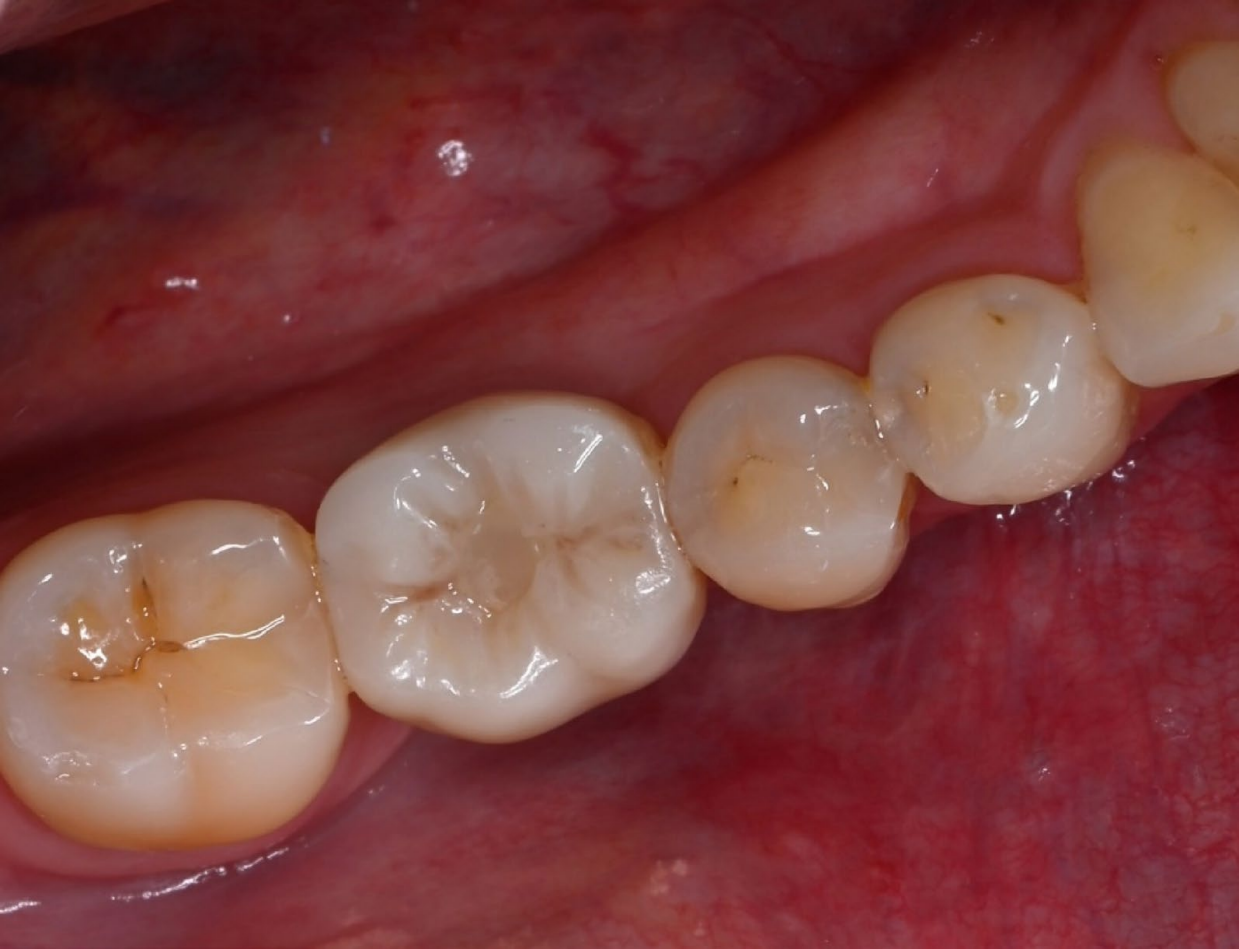
PICN-ISC in FDI position 46

**Fig. 2:**
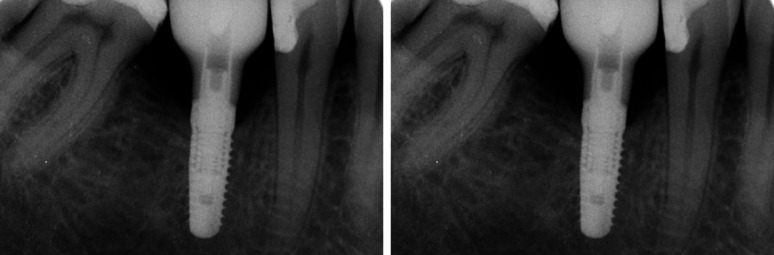
Radiographs of a ZLS-ISC at prosthetic delivery (left) and follow-up (right)

**Fig. 3:**
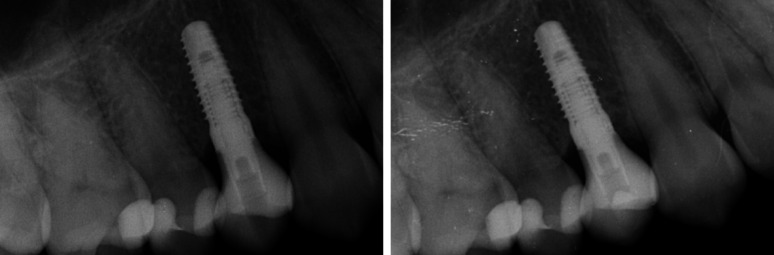
Radiographs of a PICN-ISC at prosthetic delivery (left) and follow-up (right)

Regarding occlusal contacts, at least one point-shaped contact was required for static occlusion in posterior crowns, while dynamic contacts were removed when possible. For anterior ISCs, static contacts were removed, but anterior canine guidance was preserved. Occlusion foil (12µ) and shimstock foil (8µ) were used for occlusal assessment. The ISC was retained using a new screw and the respective torque wrench according to manufacturer’s instructions (BLT: 35 Ncm, PP: 20 Ncm). Composite (Tetric EvoCeram, Ivoclar Vivadent GmbH, Germany) was used to cover the screw channel. After prosthetic delivery (Fig. 1), intraoral scans and radiographs were taken (Figs. 2 and 3), and the composition of the opposing teeth and implant distribution were reported (Tables 1 and 2).

**Table 1:** Composition of the two opposing teeth of each ISC

Opposing teeth	n
Enamel	4
Enamel + ceramic restoration	6
Enamel + metal restoration	0
Enamel + composite restoration	22
Metal restoration + metal restoration	3
Metal restoration + ceramic restoration	1
Metal restoration + composite restoration	0
Ceramic restoration + ceramic restoration	1
Ceramic restoration + composite restoration	5

**Table 2:** Distribution of ISCs according to the FDI scheme

Implant Site	PICN	ZLS	Total
Anterior	n = 2	n = 1	n = 3
Posterior	n = 29	n = 30	n = 59

PICN, polymer-infiltrated ceramic network; ZLS, zirconia-reinforced lithium silicate ceramic

### Follow-up examination

Approximately 12 months after prosthetic delivery, patients were examined clinically, and x-rays were taken (Figs. 2 and 3). Clinical evaluations included an optical examination of the restoration surface at five times magnification. Intraoral scans and photographs were taken. ISCs were not further adjusted occlusally during the follow-up period, except for the time of prosthetic delivery, to avoid influences on abrasion analysis. Due to the ongoing COVID-19 pandemic, 12-month-follow-ups could not be scheduled at the exact time required or had to be canceled. A restoration was considered as survived if it was in situ and in function at the follow-up visit. In case of unrecoverable failures (i.e. ISC fractures), a new ISC was delivered, and the patient was excluded from this investigation. Any technical complications, such as chipping, screw loosening or loss of retention, were documented.

### Occlusal wear analysis

**Fig. 4:**
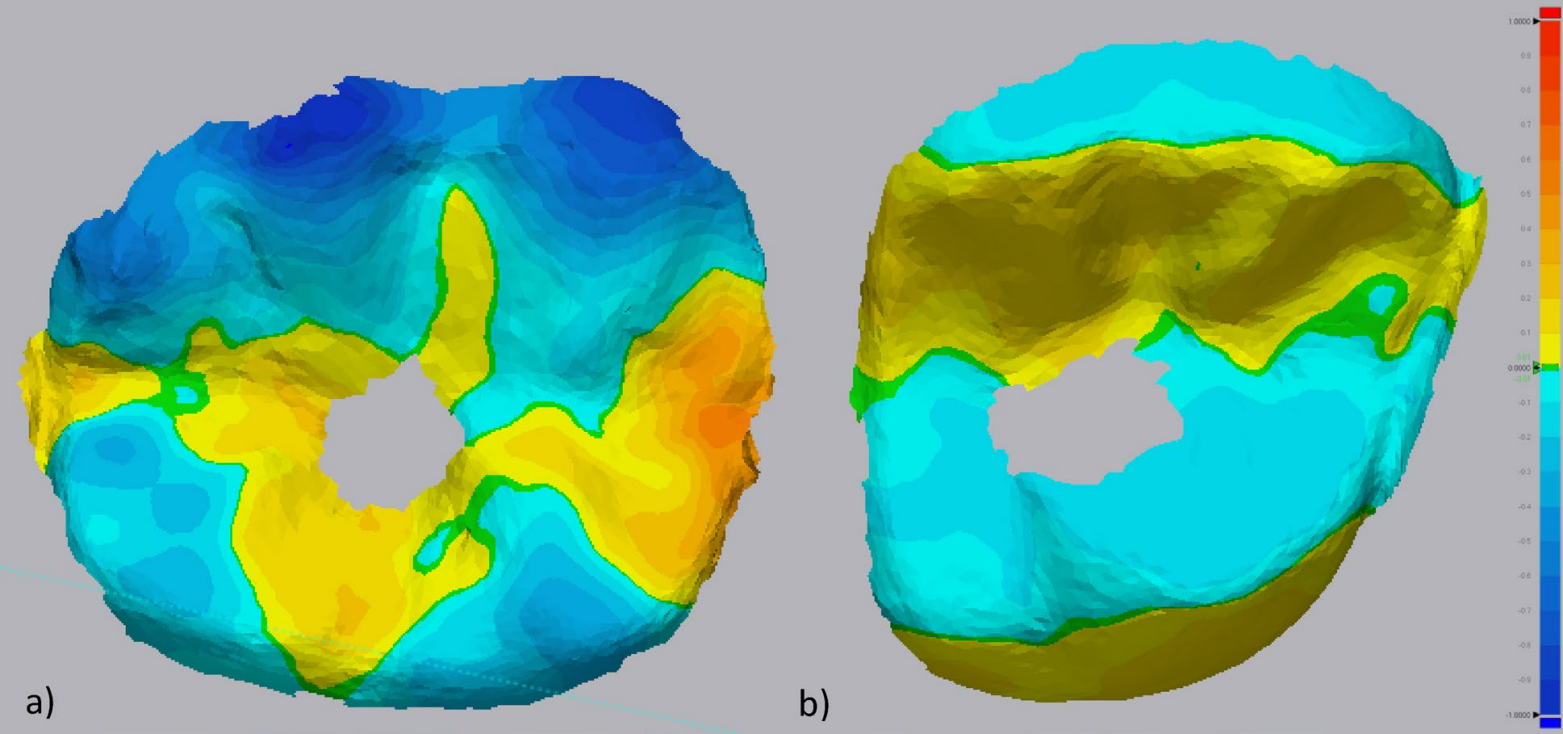
Superimposition of an exemplary PICN-ISC (**a**) and ZLS-ISC (**b**) The screw-channel area was not included in the analysis (grey), since it might have been replaced during function. (green = no difference, blue = wear). Yellowish areas might be the result of swelling (PICN) or micromovements (ZLS)

Occlusal wear of the ISCs and adjacent teeth was evaluated as a secondary outcome, utilizing a fully digital workflow [28, 29]. Maximum wear depth was calculated by superimposing intraoral scans taken at the day of prosthetic delivery (after definitive intraoral adjustments) with those obtained at the 12-month follow-up. Superimposition was performed with a 3D inspection software (Geomagic Control X, 3D Systems, USA). The standard tessellation language (STL) dataset from the time of prosthetic delivery served as a reference. After initial alignment through the inspection software, an additional refinement was conducted by using a best fit algorithm. To increase the accuracy of superimposition, only the segmented adjacent teeth were used as reference structures [30]. Soft tissues were excluded from the matching procedure to avoid possible inaccuracies. After the alignment, the maximum wear depth (in mm) of the ISC was measured with the software´s 3D comparison tool (Fig. 4). To ensure that observed wear on the ISC was not part of a broader wear pattern affecting the patient’s dentition, adjacent teeth were also analyzed for occlusal wear. The composite material covering the screw access channel was excluded from the analysis. Antagonist wear was not analyzed.

### Statistical analyses

Data were analyzed using R 3.0 (R Foundation for Statistical Computing, Austria). Descriptive data were presented as percentages. Cox proportional hazards modelling was used to identify exposures associated with the prosthetic survival and technical complication rate. Age, jaw, implant system, position, diameter, length, and material were considered as influence variables. Kaplan–Meier survival analysis was conducted to evaluate the influence of restoration material and implant system. The probability level for statistical significance was set at *p* = *0.05*.

Wear values were converted into absolute values. Data showed adherence to the normal curve (*p* > *0.05*) and was presented as mean ± standard deviation (SD). A T-test for independent samples was used to compare the wear values of both materials.

## Results

### Baseline data

Sixty-one patients aged between 23 and 82 years (mean age: 48.8 ± 14.2 years) received a single implant, and one participant was treated with two implants. Implants were evenly distributed between mandible and maxilla (Table 3). Three implants (4.8%) were placed in the anterior region, while 59 (95.2%) were in posterior sites. Seven implants (11.3%) showed a diameter of < 4 mm, while 55 implants (88.7%) had a diameter of > 4 mm.

**Table 3:** Distribution of evaluated implants according to location (maxilla/mandible), diameter (< 4 mm/ > 4 mm) and implant type (BLT/PP)

Location	Diameter	Implant type
Maxilla n = 22	< 4.0 mm n = 5	BLT n = 25
Mandible n = 20	> 4.0 mm n = 37	PP n = 17

**Fig. 5:**
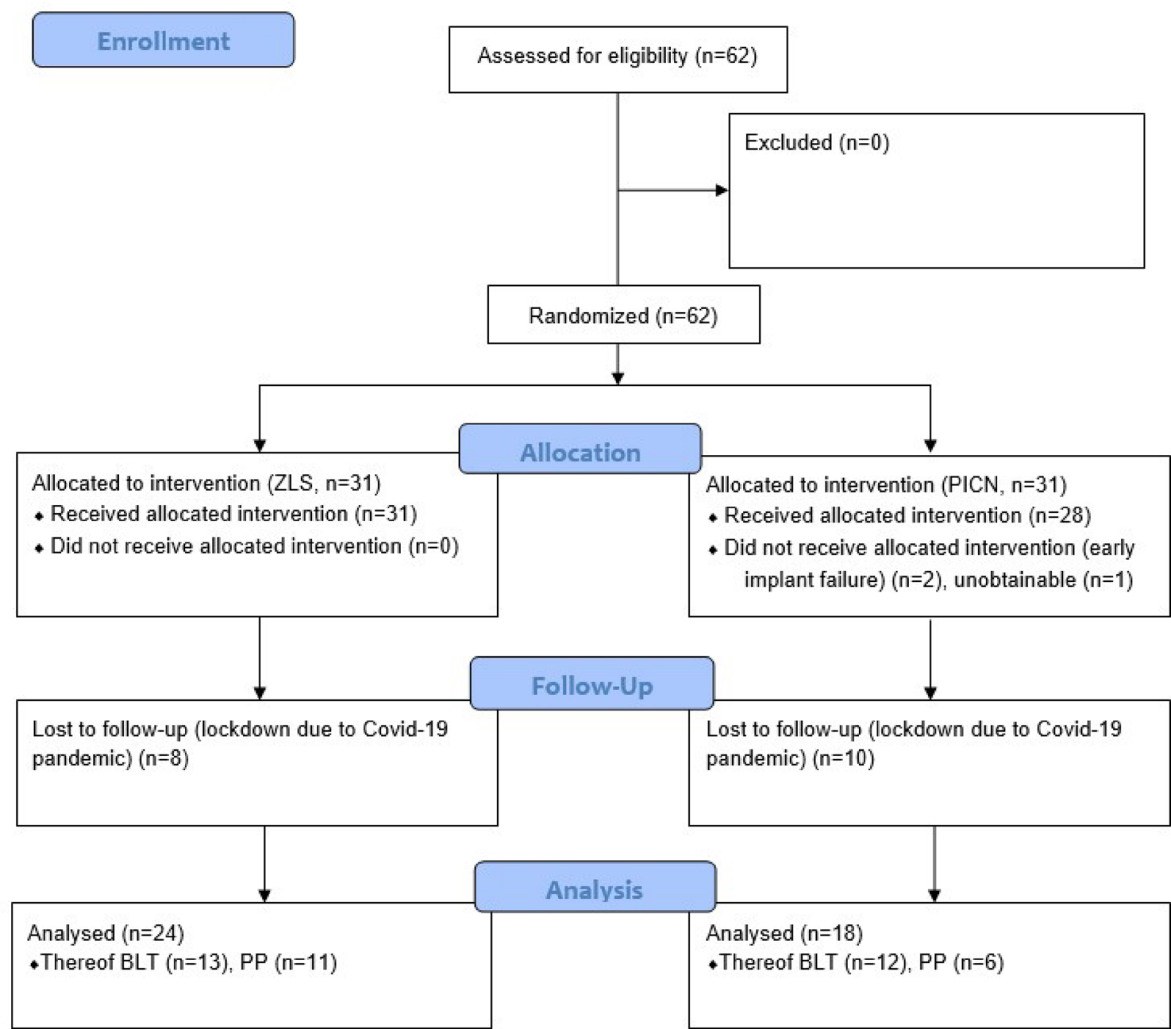
Flowchart describing the two cohorts from the randomization procedure to the 12-month follow-up

From sixty-one recruited patients, eighteen were lost to follow-up at the 1-year mark, primarily because of the restrictions due to the ongoing Covid-19 pandemic, which imposed a curfew. Exceptions were only possible for essential medical reasons or emergencies, which the conduction of this study was not a part of. As a result, patients were not allowed to attend the scheduled follow-up appointments. In addition, two implants (BLT n = 1; PP = 1) failed prior to prosthetic delivery. As a result, forty-two dental implants-supported restorations were evaluated (PICN n = 18; ZLS n = 24), as shown in Fig. 5. The mean observation time was 409.5 ± 102.6 days.

### Survival rate

**Fig. 6:**
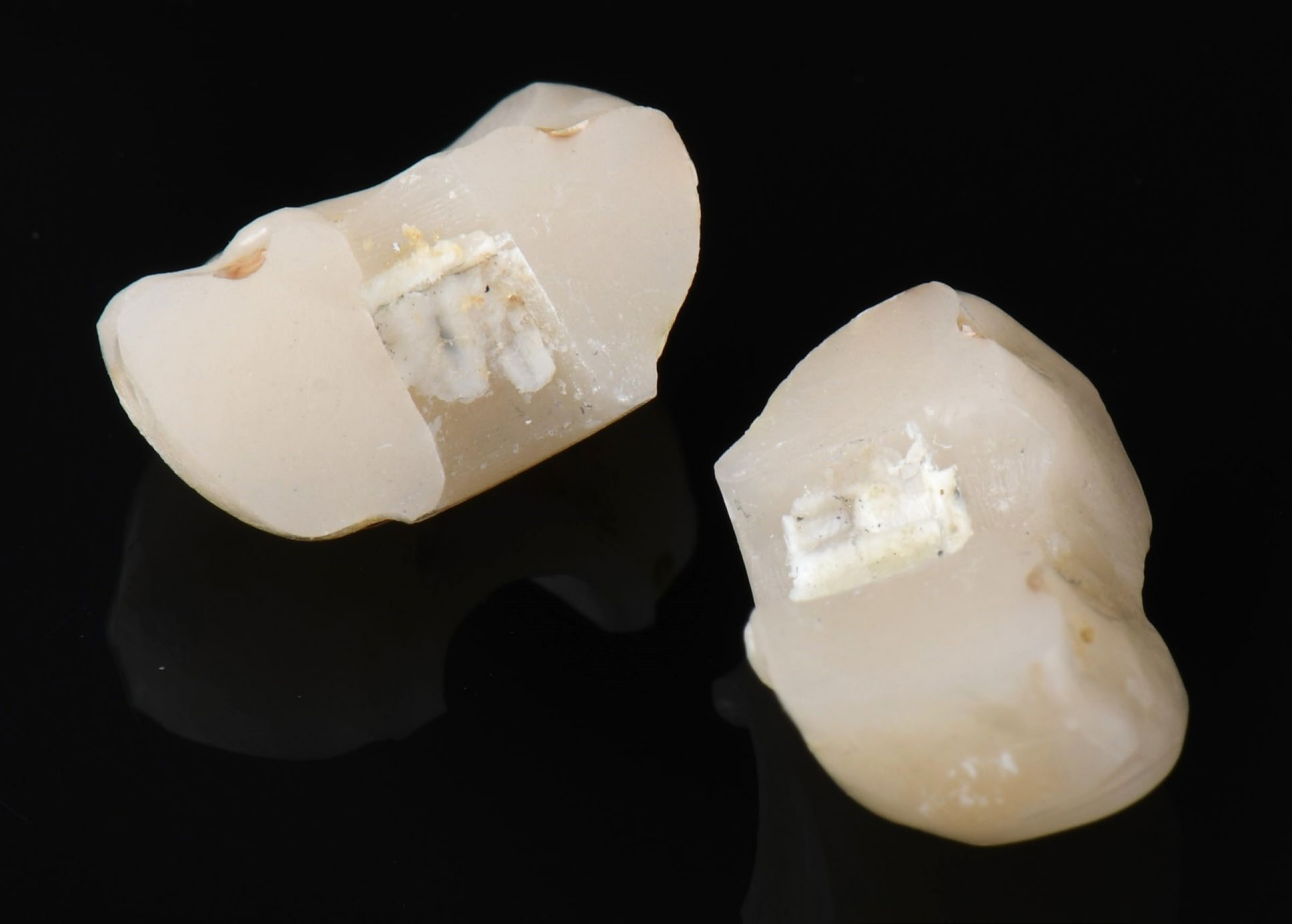
An exemplary sample of a fractured PICN-ISC with partial luting remnants (white material) inside the crown

**Fig. 7:**
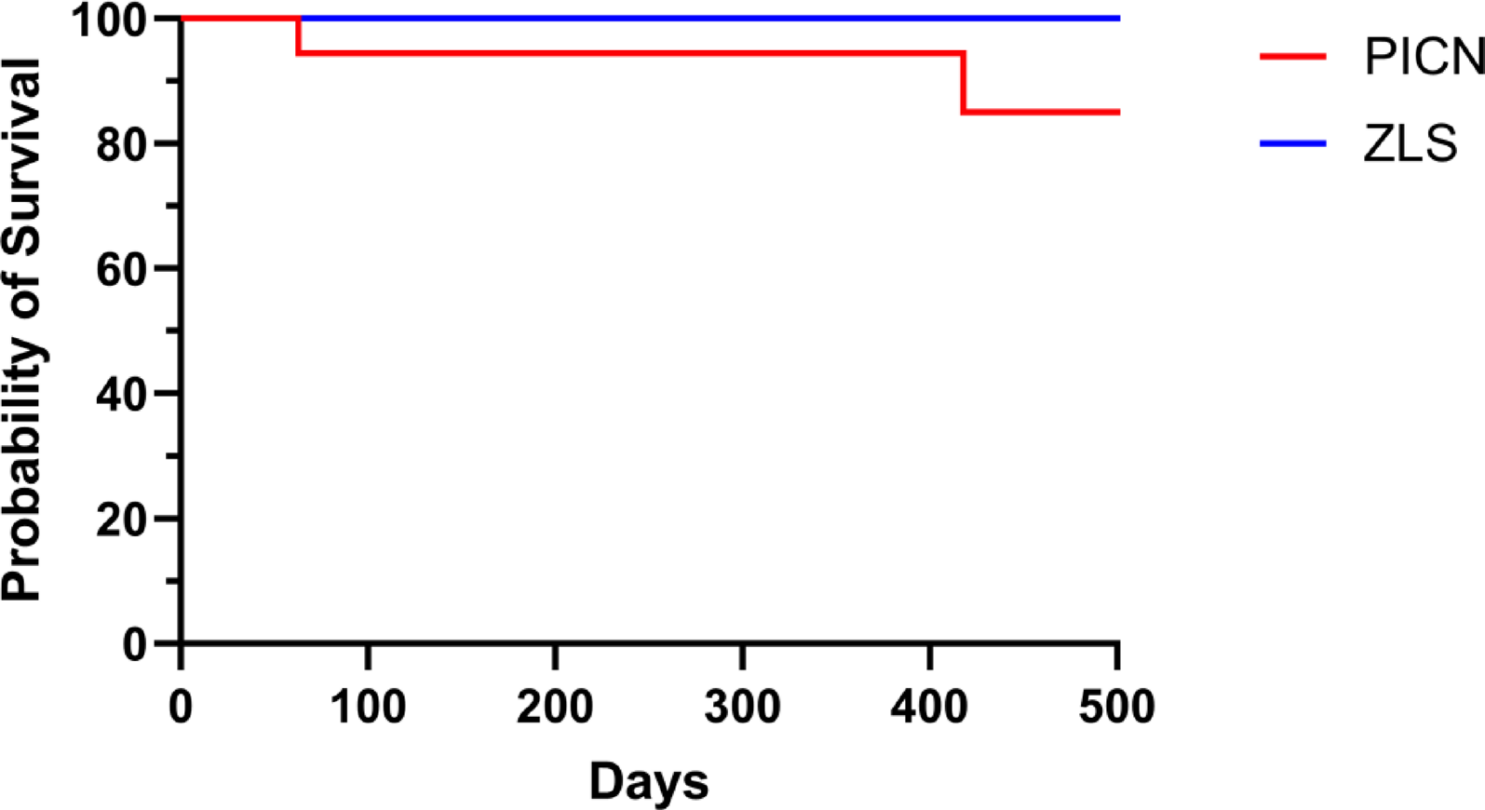
Kaplan–Meier survival curve by restoration material. (PICN, polymer-infiltrated ceramic network; ZLS, zirconia-reinforced lithium silicate ceramic)

Prosthetic survival was assessed for the included 42 ISCs. Two catastrophic crown fractures (Fig. 6) were observed, both occurred in the PICN group, leading to a survival rate of 84% for this material (Table 4). Differently, no catastrophic fractures were observed for ZLS, yielding a 100% survival rate. However, the difference in survival rates between PICN and ZLS did not reach statistical significance (P = 0.16) (Fig. 7).

**Table 4:** Overview of prosthetic failures and survival rate depending on the material

Material	Number of prosthetic failures	Survival rate
PICN	2	84%
ZLS	0	100%

PICN, polymer-infiltrated ceramic network; ZLS, zirconia-reinforced lithium silicate ceramic

When focusing on the implant systems, no failure occurred for PP (100% survival), whereas irreparable ISC fractures occurred in the BLT group (82% survival). Nevertheless, no statistical significance was observed between the 2 implant systems (P = 0.13). Other potential influencing factors—such as patient age (P = 0.23), jaw (P = 0.85), implant site (anterior vs. posterior; P = 1.00) and diameter (< 4 mm vs. > 4 mm; P = 1.00)—also did not show statistically significant effects on prosthetic survival. All failures were associated with titanium bases of 3.5 mm in height and Ø 4.5 mm, but no significant correlation was found between titanium base height and failure rate (P = 0.99).

Circumferential material thickness of both failed crowns was at least 1 mm (manufacturer’s recommendation: 0.8 mm).

### Technical complications

**Fig. 8:**
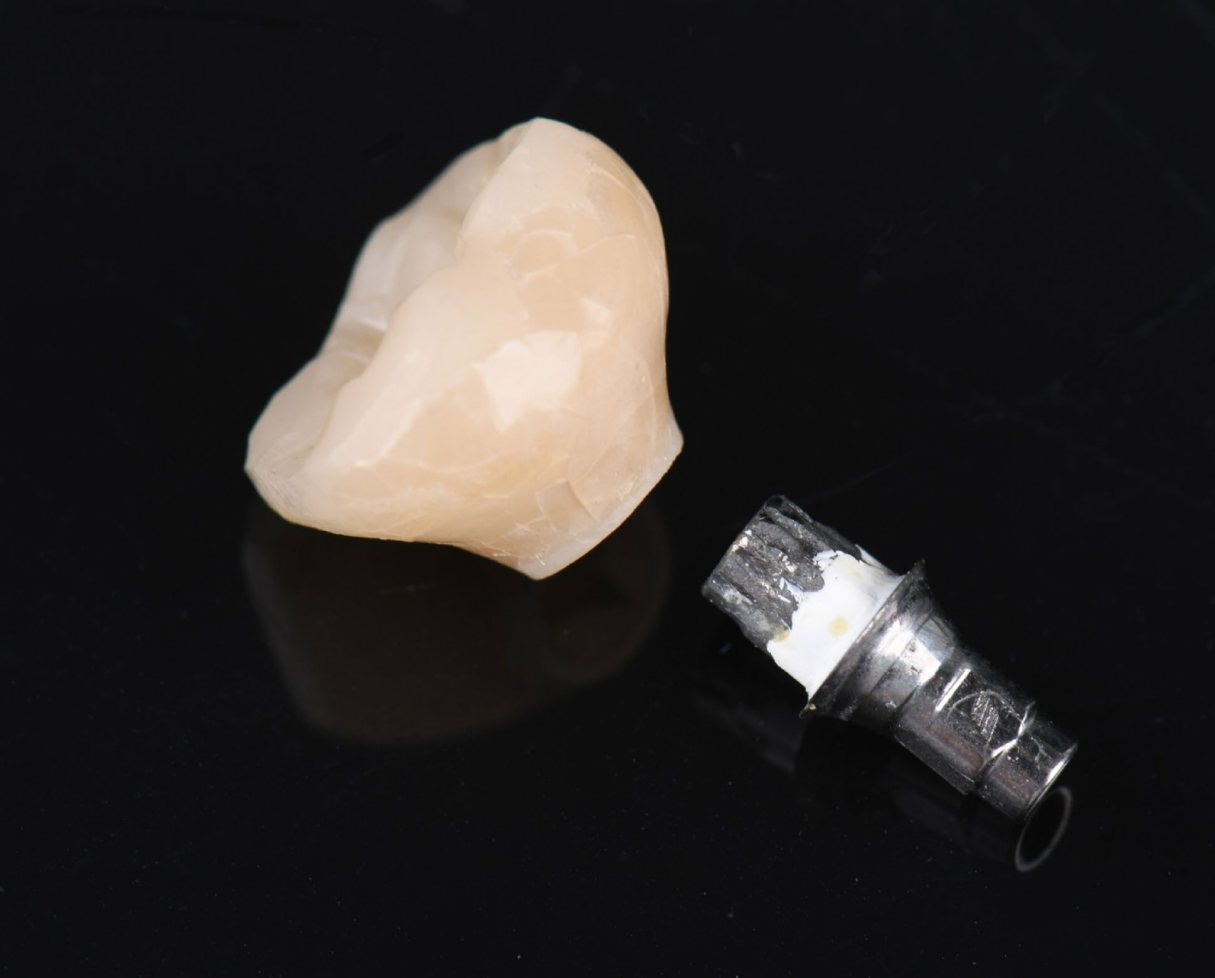
An example of debonded PICN-ISC and titanium base. Cement remnants are clearly visible on the cervical abutment surface, and numerous fracture lines are visible on the basal part of the crown surface

**Fig. 9:**
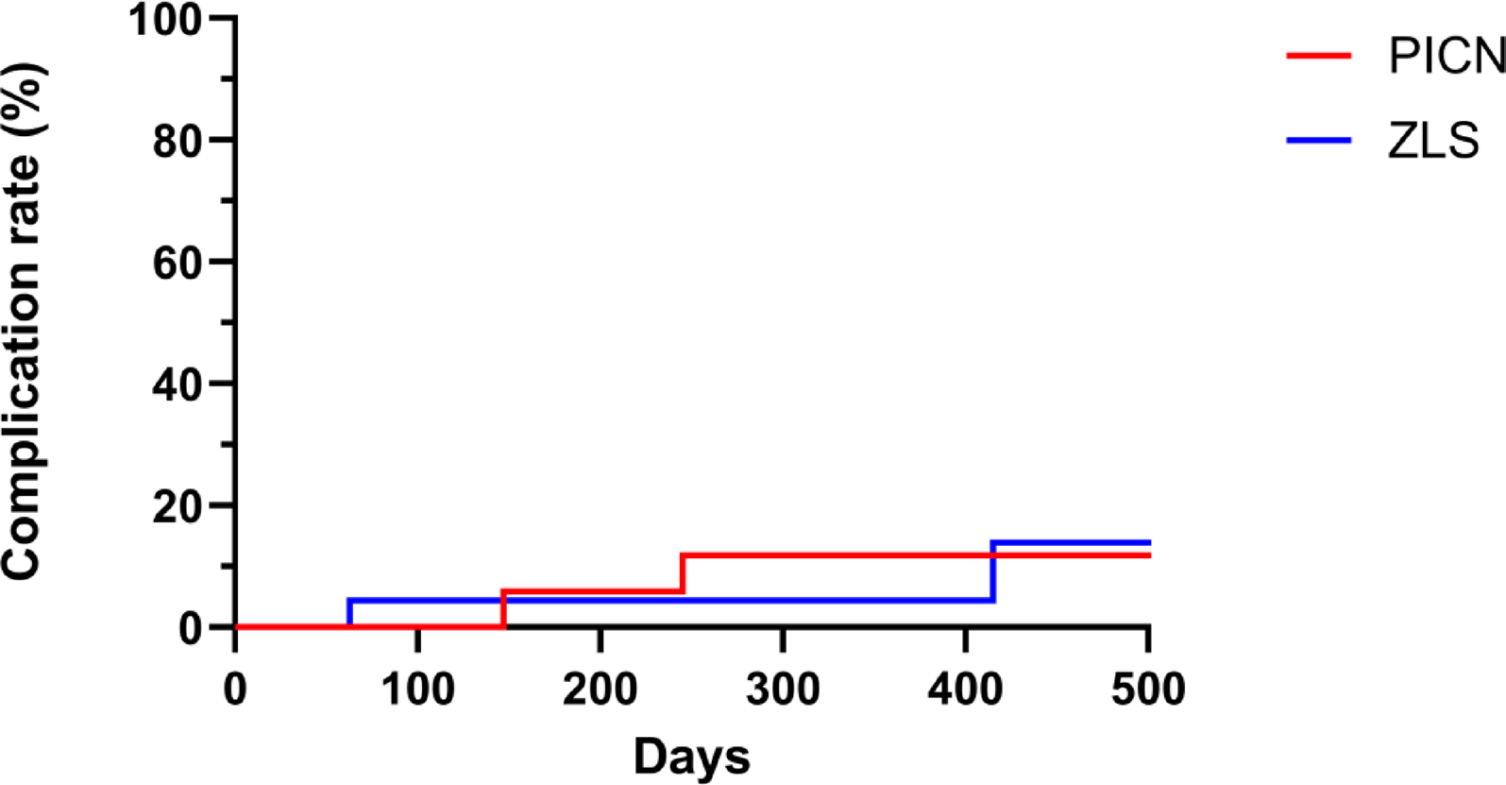
Kaplan–Meier curve of technical complication rate. (PICN, polymer-infiltrated ceramic network; ZLS, zirconia-reinforced lithium silicate ceramic)

In five patients, debonding from the titanium base was observed, as illustrated in Fig. 8. This complication occurred in 2 PICN (14% complication rate) and 3 ZLS restorations (15% complication rate; Fig. 9 and Table 5). The difference in terms of debonding rate between the 2 materials did not reach statistical significance (P = 0.72). When focusing on the implant type, no statistical significance between the systems was observed (P = 0.15). Additionally, the occurrence of technical complications was analyzed in relation to titanium base height, but no statistically significant correlation was identified (P = 0.99). No other technical complications were reported.

**Table 5:** Overview of technical complications depending on the material

Material	Loss of retention	Screw loosening	Chipping
PICN	2	0	0
ZLS	3	0	0

PICN, polymer-infiltrated ceramic network; ZLS, zirconia-reinforced lithium silicate ceramic

### Occlusal wear

**Fig. 10:**
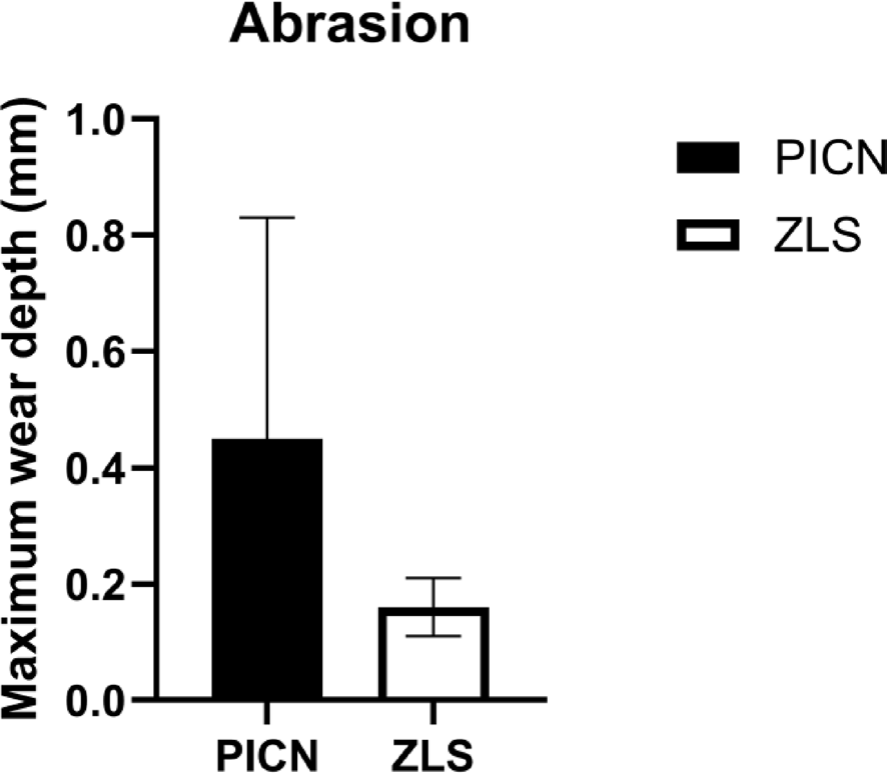
Box plot showing the difference in occlusal wear in mm between PICN (black) and ZLS (white). (PICN, polymer-infiltrated ceramic network; ZLS, zirconia-reinforced lithium silicate ceramic)

Occlusal wear was assessed for 21 ISCs (PICN: n = 10; ZLS: n = 11) (Table 6). Significantly greater maximal occlusal wear was reported in the PICN group (0.45 mm ± 0.38 mm) compared to the ZLS group (0.16 mm ± 0.05 mm; P = 0.03) (Fig. 10).

**Table 6:** Number of ISCs evaluated for abrasion according to FDI-position

2	3	/	4	/	/	/	/	/	/	1	/	1	1
17	16	15	14	13	12	11	21	22	23	24	25	26	27
47	46	45	44	43	42	41	31	32	33	34	35	36	37
/	2	/	/	/	/	/	/	/	/	/	/	7	/

PICN-based ISCs showed significantly higher wear compared to their adjacent teeth (0.13 mm ± 0.04 mm; P = 0.01), whereas no statistically significant difference was found between the ZLS-ISCs and their adjacent teeth (0.18 mm ± 0.08 mm; P = 0.51) .

The composition (natural tooth, composite, ceramic, metal) and type of opposing dentition (premolar/premolar, premolar/molar, molar/molar) were considered as covariables. Among these, only the molar/molar combination showed a statistically significant effect (P = 0.04).

## Discussion

To the best of the authors’ knowledge, this is the first clinical study comparing PICN- and ZLS-based ISCs. The results show higher complication and failure rates, when compared to data from the literature on monolithic ISCs made from zirconia (ZrO_2_) or lithium disilicate (LiSi_2_) [7]. Two implants (BLT n = 1; PP n = 1) were lost in total, both before prosthetic delivery. The pre-loading implant failure might be related to the clinicians' learning curve or to undetected anatomical or medical reasons [31].

The null hypothesis assumed at study conceptualization was rejected. Although, similar survival and complication rates were observed for PICN- and ZLS-based ISCs, occlusal wear was significantly higher for PICN.

Previous in-vitro studies have reported the influence of titanium base height on fracture resistance and retention of ISCs [32–35]. Both failed crowns presented a 3.5 mm height titanium base. Noteworthily, the manufacturer of the evaluated PICN recommends using titanium bases at least 4 mm high. This is challenging in clinical situation with reduced vertical prosthetic space when anatomical limitations do not allow subcrestal implant installation. Particularly when restoring a BLT implant, the corresponding RC Variobase (Institut Straumann AG, Switzerland) was only available in 3.5 mm and 5.5 mm height. In both failed ISCs, choosing the 5.5 mm titanium base, would have meant to undercut the minimum occlusal material thickness of 1 mm. The use of titanium bases with a reduced height of 3.5 mm may also have contributed to the fact, that both fractures were observed in the group of BLT implants. Given the limited number of catastrophic failures and the relatively small sample size, implant-system–related differences should be interpreted cautiously.

Previous clinical studies evaluating PICN-ISCs do not report details on the titanium base height used [36]. Still, this might be a key factor contributing to the survival and technical complication rate of PICN-ISCs. In addition, achieving the necessary circumferential material thickness at titanium base level, might not be possible in cases where a narrow emergence profile is desirable [37]. Future trials should report detailed information regarding the titanium base conformation and luting protocol.

In the present study, debonding between ISCs and the titanium base was the only technical complication, observed in five cases (PICN n = 2; ZLS n = 3), occurring at considerably higher rates than reported for monolithic ZrO₂ and LiSi₂ restorations [1]. This observation is consistent with the findings of a recently published clinical investigation, in which debonding from the titanium base was likewise reported as the most frequent complication for the same PICN material of this study [20].

Still, prospective clinical trials comparing outcomes of PICN with other materials are scarce. A clinical investigation reported a 70% 5-year survival rate for ISCs made from Lava Ultimate (3 M Oral Care, USA) [38]. In another study, Lohbauer et al. identified adhesive failure between crown and abutment as the main cause of ISC fracture, in restorations made from the same material [39]. A comparable failure mechanism may also have occurred in the present study, as remnants of luting material were found predominantly on the titanium base surface. Burkhardt et al. further demonstrated, that pull-off forces vary considerably depending on the bonding system used [40]. The combination applied in the present study—PICN with Multilink Hybrid Abutment (Ivoclar Vivadent GmbH, Germany)—showed significantly lower values (367 N) compared with PICN bonded with Panavia V5 (Kuraray Noritake Dental Inc., Japan), which achieved 545 N [40]. These results indicate that failure and complication rates might be reduced depending on the adhesive protocol applied. Furthermore, the impact of parameters such as crown design and abutment dimension should be further evaluated. Another possible reason for ISC failure can be related to the microscopic characteristics of PICN. Swelling in the basal part of the ISC due to water absorption, as previously reported by Lohbauer et al., might cause crack formation and propagation (Figs. 6 and 8). As a consequence, debonding episodes occur from the most cervical part of the titanium base-ICS-interface with successive stress increasing in the luting layer and finally leading to failure. Swelling due to water absorption of the luting agent itself could decrease bonding strength and be a further reason for failure [41–43]. ZLS on the contrary does not contain polymers and so might not be susceptible to this phenomenon. No screw loosening occurred, likely due to the reliable implant–abutment connections and adapted occlusal scheme, with PP implants using a butt-joint and BLT implants an internal conical connection. The use of a new screw at prosthetic delivery and strict adherence to the manufacturer's torque recommendations may also have contributed to this outcome.

Chipping episodes were not observed, while occlusal wear was significantly higher for PICN compared to ZLS. This may be attributed to differences in microstructure. It should be noted however, that only a limited number of ISCs could be included in the wear analysis. Additional data, particularly including opposing dentition, are needed.

Different methods for wear analysis are described in the literature [28, 29, 44, 45]. In the present study a fully digital workflow was applied to simplify and standardize the experimental process and to minimize inaccuracies related to conventional impressions, stone cast fabrication, and subsequent digitization steps. Intraoral scans were obtained at prosthetic delivery and at follow-up using the same scanner system, and occlusal wear was quantified by superimposition of STL datasets using segmented adjacent teeth as reference structures, while excluding soft tissues. This approach was chosen to reduce cumulative errors associated with material distortion, cast expansion, trimming inaccuracies, and scanning of physical models, which have been reported to influence wear measurements in conventional workflows [45]. At the same time, it must be acknowledged that the simplification and standardization achieved through a fully digital workflow may also have influenced the experimental results. Intraoral scanning accuracy, alignment procedures, and best-fit algorithms are subject to inherent limitations and may introduce systematic deviations, particularly when small dimensional changes are analyzed over time. The yellow areas visible in the superimposition maps (Fig. 4) may therefore represent true material-related changes, such as swelling-related expansion in PICN-ISCs, but may also partly reflect minor inaccuracies related to scan matching and alignment. Furthermore, despite the exclusion of soft tissues and the use of adjacent teeth as reference structures, subtle changes in tooth position or wear of reference surfaces cannot be entirely excluded.

Overall, the unified digital workflow proved advantageous in terms of feasibility, reproducibility, and reduction of analog-related variability, especially under clinical conditions. However, the wear data should be interpreted with caution, as the applied methodology may have affected the absolute magnitude of the measured wear values.

Clinical evaluations of occlusal wear with PICN- and ZLS-ISCs are scarce. In-vitro studies have shown comparable wear values for PICN (0.31 ± 0.04 mm), though, comparisons between preclinical and clinical scenarios should be interpreted cautiously [46]. In this study, statistically significant differences between wear values for ZLS and PICN were reported. ZLS exhibited a mean maximal occlusal wear of 0.16 mm ± 0.05 mm, whereas PICN showed higher and more variable wear of 0.45 mm ± 0.38 mm. Abrasion values recorded for ZLS are comparable to data from the literature on ZrO2, and LiSi [47, 48]. Maximal wear observed for both materials, did not result in prosthetic or biological problems in the present study and can therefore be regarded as clinically acceptable. However, considering the short follow-up period, greater maximal wear recorded for PICN, might become clinically relevant over extended service periods.

Wear evaluation of the opposing dentition was not performed, which is a limitation of this study. A further limitation of this study is the absence of a control group such as LiSi or ZrO_2_ ISCs, two of the most studied materials for this indication. Including such a control group would have strengthened the study design and helped eliminate certain bias from operator or laboratory factors. Additionally, biological parameters like bleeding on probing, marginal bone loss or influence of emergence profile design were not evaluated. Furthermore, parafunctional habits such as bruxism and the use of occlusal protective appliances were not systematically assessed, which may have influenced both technical complication and wear rates [49]. The number of evaluated crowns (n = 42) compared to the ones delivered was relatively low, which may introduce potential selection bias and limits the statistical power. Nevertheless, baseline characteristics of patients who did not complete the follow-up were comparable, suggesting that the impact on the study’s conclusions is likely minimal. Considering the short follow-up period, the presented data indicates the risk for early drawbacks of the investigated ISC systems and should be further analyzed.

## Conclusions

Regardless of the implant system, ZLS exhibited the highest prosthetic survival with no catastrophic failures, while PICN showed lower survival due to irreparable crown fractures. Although the differences in survival between materials, implant types, and patient- or implant-related variables did not reach statistical significance, all failures were associated with 3.5 mm titanium base height. Technical complications, particularly loss of retention from the titanium base, occurred at similar rates in both material groups and were not influenced by implant system or abutment height. In contrast, occlusal wear revealed a distinct material-dependent effect, with PICN showing significantly higher wear than ZLS and adjacent teeth, especially in molar-to-molar occlusion.

## Data Availability

The datasets used and analyzed during the current study are available from the corresponding author on reasonable request.
